# Oxygen Therapy Use in Older Adults with Chronic Obstructive Pulmonary Disease

**DOI:** 10.1371/journal.pone.0120684

**Published:** 2015-03-18

**Authors:** Shawn P. E. Nishi, Wei Zhang, Yong-Fang Kuo, Gulshan Sharma

**Affiliations:** 1 Division of Pulmonary Critical Care & Sleep Medicine, Department of Internal Medicine, University of Texas Medical Branch, Galveston, Galveston, Texas, United States of America; 2 Sealy Center on Aging, University of Texas Medical Branch, Galveston, Galveston, Texas, United States of America; University of Dundee, UNITED KINGDOM

## Abstract

**Rationale:**

Oxygen therapy improves survival and function in severely hypoxemic chronic obstructive pulmonary disease (COPD) patients based on two landmark studies conducted over 40 years ago. We hypothesize that oxygen users in the current era may be very different. We examined trends and subject characteristics associated with oxygen therapy use from 2001–2010 in the United States.

**Methods:**

We examined Medicare beneficiaries with COPD who received oxygen from 2001 to 2010. COPD subjects were identified by: 1) ≥2 outpatient visits >30 days apart within one year with an encounter diagnosis of COPD; or 2) an acute care hospitalization with COPD as the primary or secondary discharge diagnosis. Oxygen therapy and sustained oxygen therapy were defined as ≥1 and ≥11 claims for oxygen, respectively, in the durable medical equipment file in a calendar year. Primary outcome measures were factors associated with oxygen therapy and sustained oxygen therapy over the study period.

**Results:**

Oxygen therapy increased from 33.7% in 2001 to 40.5% in 2010 (p-value of trend <0.001). Sustained oxygen therapy use increased from 19.5% in 2001, peaked in 2008 to 26.9% and declined to 18.5% in 2010. The majority of subjects receiving oxygen therapy and sustained oxygen therapy were female. Besides gender, factors associated with any oxygen use or sustained oxygen therapy were non-Hispanic white race, low socioeconomic status and ≥2 comorbidities.

**Conclusions:**

Any oxygen use among fee-for service Medicare beneficiaries with COPD is high. Current users of oxygen are older females with multiple comorbidities. Decline in sustained oxygen therapy use after 2008 may be related to reimbursement policy change.

## Introduction

Oxygen therapy improves survival of chronic obstructive pulmonary disease (COPD) patients with hypoxemia [1, 2]. Other benefits of oxygen therapy include improvements in sleep, cognitive function, emotional status and slowing the progression of hypoxic pulmonary hypertension [1–4]. In the United States, over 1.4 million Medicare patients in 2008 received oxygen therapy at an estimated cost of $2.9 billion which accounted for more than 45% of Medicare durable medical equipment (DME) expenditure that year. Of these, 82% have a diagnosis of COPD [5, 6].

Prescription of oxygen therapy is predicated on two small landmark randomized controlled studies conducted over 40 years ago—British Medical Research Council (MRC) and the National Heart, Lung, and Blood Institute (NHLBI) Nocturnal Oxygen Therapy Trial (NOTT) [1, 2]. Medicare qualifying criteria are based on these trials: 1) Oxygen saturation ≤88% or PaO2 ≤55 mmHg; or 2) SpO2 ≤89% or PaO2 56–59 mmHg and with either a) dependent edema suggesting congestive heart failure, b) pulmonary hypertension or cor pulmonale or c) hematocrit ≥55%[7]. Clinical practice guidelines focus on oxygen therapy benefits among: 1) *stable* COPD patients with severe resting hypoxemia, 2) non-smokers, and 3) oxygen use ≥15 hours daily [8, 9]

Subjects enrolled in these studies were primarily non-Hispanic white males, median age <65 years and without significant comorbidities. The current COPD population is very different than those studied in the landmark trials. There are no national studies describing the current use of any oxygen therapy or sustained oxygen therapy among COPD patients in the United States. Using a 5% national sample of fee-for-service Medicare beneficiaries, we examined national trends and factors associated with the use of oxygen therapy and sustained oxygen therapy in older adults with COPD between 2001 and 2010.

## Methods

### Data Source

This is a retrospective study of oxygen therapy use in subjects with COPD using a 5% fee-for-service Medicare beneficiary population from 2001–2010. This study was approved by the University of Texas Medical Branch Institutional Review Board and informed consent was not obtained due to the nature of the study. All records were de-identified prior to analysis. Data from the following files were used for this study: 1) Denominator File (Medicare enrollment information and demographic data); 2) Medicare Provider Analysis and Review file (claims for hospital inpatient and skilled nursing facility stays); 3) Outpatient Standard Analytic File (hospital outpatient services); 4) 100% Physician/Supplier File (physician and other medical services); and 5) Durable Medical Equipment (DME) File [10].

### Study Cohort

A separate denominator file of beneficiaries with COPD was created for each calendar year (2001–2010). Each file was composed of participants in the year of interest who: 1) had a diagnosis of COPD in the year; 2) was aged ≥66 years; 3) had complete enrollment (Part A, Part B) in the previous and current calendar year or until death; 4) were not enrolled in a health maintenance organization (HMO); and 5) were not a resident of a nursing facility in the previous and current calendar year or until death.

A patient met the diagnosis of COPD who had any of the following: 1) two or more outpatient visits at least 30 days apart within one year noted by Evaluation and Management (E&M) codes 99201–99205 or 99211–99215 with an encounter diagnosis of COPD based on International Classification of Diseases Ninth Revision (ICD-9) codes 491.x, 492.x, or 496.x; or 2) an acute care hospitalization with COPD being the primary discharge diagnosis; or 3) an acute care hospitalization for respiratory failure (ICD-9 codes 518.81, 518.82, or 518.84) as the primary discharge diagnosis and COPD listed as the secondary diagnosis. Oxygen therapy use was defined from Healthcare Common Procedure Coding System (HCPCS) codes E1390-E1392.

### Variables

Subject demographic characteristics included the following: age, gender, race, number of comorbidities [11, 12] and United States geographic region. Socioeconomic status was based on eligibility for at least one month during the index year for state buy-in coverage provided by the Medicaid program. We defined oxygen use as: any oxygen use and sustained oxygen as ≥1 and ≥11 claims for oxygen in a calendar year, respectively.

### Assessment of Model of Care

Outpatient physician, primary care physician (PCP) and pulmonary specialist visits during the 365 days prior to the index date were calculated. PCPs include physicians in any of the following specialties: family medicine, general practice, internal medicine and geriatrics. Beneficiaries who had a PCP visit and a pulmonary physician visit within one year prior to the index date were considered as being co-managed; non-PCP or non-pulmonary physicians were considered as “other” physician. Pulmonary rehabilitation use was identified through the HCPCS codes G0237, G0238, or G0239 or Common Procedural terminology (CPT) codes 97001, 97003, 97110, 97116, 97124, 97139, 97150, 97530, 97535, or 97537 with primary ICD-9 codes 491.x, 492.x, or 496.x.

### Statistical Analysis

The proportion of subjects who received oxygen therapy and sustained oxygen therapy by year were calculated and plotted by age, gender, race, and comorbidity. Patient characteristics were summarized using counts and percentages of categorical variables. The chi-square test was used to compare patient characteristics of those who did and did not use oxygen therapy and sustained oxygen therapy. A generalized estimate equation model was used to adjust for clustering at patient level to assess the trend in oxygen therapy and sustained oxygen therapy. Sensitivity analyses were performed for subjects with ≥1, ≥6 and ≥11 oxygen claims by calendar year and by rolling calendar months. We also examined pulmonary rehabilitation use and computed median survival by gender among sustained oxygen therapy users. All analyses were performed using SAS version 9.2 (SAS Inc., Cary, NC). All reported p-values were two-sided with p<0.05 considered statistically significant.

## Results

Table 1 presents the characteristics of Medicare beneficiaries with COPD and percent receiving any oxygen therapy and sustained oxygen therapy from 2001 to 2010. Over the 10 year study period, there were 329,482 COPD subjects with an average age of 75.8 years old. Of these, 128,300 (38.9%) received oxygen therapy and 73,659 (22.4%) received sustained oxygen therapy. Regional variations in use of oxygen therapy and sustained oxygen therapy were similar with lowest claims in both in the New England (30.3% and 16.9%, respectively) and Middle Atlantic regions (29.6% and 16.9%, respectively) and highest use in the Mountain region (53.2% and 32.2%, respectively). The majority of subjects were non-Hispanic white, <85 years and were not of low socioeconomic status. Additionally, more than half of beneficiaries had a comorbidity score ≥2 and use of oxygen therapy and sustained oxygen therapy increased with the number of comorbidities.

**Table 1:** Comparison of baseline characteristics of Medicare beneficiaries on Oxygen Therapy with Chronic Obstructive Pulmonary Disease (COPD) ^1^, 2001–2010.

		Any Oxygen Use		Sustained Oxygen Use^2^	
Characteristic	Overall	Yes	P	Yes	P
		N(%)	Value	N(%)	Value
**All subjects**	329,482	128,300(38.9)		73,659 (22.4)	
**Age**			0.0002		<.0001
66–74	152,003 (46.1)	58,901 (38.8)		34,422 (22.7)	
75–84	143,171 (43.5)	56,269 (39.3)		32,285 (22.6)	
≥85	34,308 (10.4)	13,130 (38.3)		6,952 (20.3)	
**Gender**			<.0001		<.0001
Female	169,901 (51.6)	69,371 (40.8)		40,794 (24.0)	
Male	159,581 (48.4)	58,929 (36.9)		32,865 (20.6)	
**Mean Age (yrs)**			0.2811		<.0001
	75.8 (6.3)	75.8 (6.3)		75.7 (6.2)	
**Race**			<.0001		<.0001
White	300,413 (91.2)	11,8791 (39.5)		68,185 (22.7)	
Black	17,310 (5.2)	5,946 (34.4)		3,460 (20.0)	
Other	11,759 (3.6)	3,563 (30.3)		2,014 (17.1)	
**Low Socioeconomic Status** ^3^			<.0001		<.0001
No	274,793 (83.4)	105,212 (38.3)		59,746 (21.7)	
Yes	54,689 (16.6)	23,088 (42.2)		13,913 (25.4)	
**Year**			<.0001		<.0001
2001	31,904 (9.7)	10,741 (33.7)		6,234 (19.5)	
2002	32,957 (10.0)	11,747 (35.6)		6,794 (20.6)	
2003	33,526 (10.2)	12,493 (37.3)		7,362 (22.0)	
2004	33,985 (10.3)	12,931 (38.1)		8,040 (23.7)	
2005	34,283 (10.4)	13,354 (39.0)		8,021 (23.4)	
2006	32,337 (9.8)	13,079 (40.5)		8,288 (25.6)	
2007	31,177 (9.5)	12,912 (41.4)		8,327 (26.7)	
2008	31,142 (9.5)	13,044 (41.9)		8,389 (26.9)	
2009	33,721 (10.2)	14,061 (41.7)		5,829 (17.3)	
2010	34,450 (10.5)	13,938 (40.5)		6,375 (18.5)	
**Region** ^4^			<.0001		<.0001
New England	16,818 (5.1)	5,089 (30.3)		2,840 (16.9)	
Middle Atlantic	47,647 (14.5)	14,102 (29.6)		8,057 (16.9)	
East North Central	57,105 (17.3)	23,097 (40.5)		13,262 (23.2)	
West North Central	21,127 (6.4)	9,313 (44.1)		5,476 (25.9)	
South Atlantic	77,858 (23.6)	31,503 (40.5)		18,198 (23.4)	
East South Central	27,558 (8.4)	11,313 (41.1)		6,490 (23.6)	
West South Central	34,909 (10.6)	14,389 (41.2)		8,079 (23.1)	
Mountain	16,235 (4.9)	8,631 (53.2)		5,220 (32.2)	
Pacific	30,225 (9.2)	10,863 (35.9)		6,037 (20.0)	
**Elixhauser Comorbidity Score** ^5^			<.0001		<.0001
0	76,388 (23.2)	25,885 (33.9)		14,061 (18.4)	
1	87,013 (26.4)	30,246 (34.8)		17,417 (20.0)	
≥2	166,081 (50.4)	72,169 (43.5)		42,181 (25.4)	

1. A **Chronic Obstructive Pulmonary Disease (COPD)** diagnosis is defined as having International Classification of Diseases, ninth revision (ICD-9) codes 491.x [chronic bronchitis], 492.x [emphysema], or 496 [chronic airway obstruction].

2. Sustained oxygen use is defined as ≥11 claims for oxygen therapy during the calendar year

3. **Socioeconomic status:** based on whether the patient was eligible for state buy-in coverage provided by the Medicaid program for at least one month during the index year;

4. **Region:** Geographic region was divided into 9 CMS regions;

5. **Elixhauser comorbidity** components: chronic pulmonary disease, congestive heart failure, valvular disease, pulmonary circulation disorders, peripheral vascular disorders, hypertension, paralysis, other neurological disorders, diabetes-uncomplicated, diabetes-complicated, hypothyroidism, renal failure, liver disease, peptic ulcer disease excluding bleeding, AIDS (acquired immune deficiency syndrome), lymphoma, metastatic cancer, solid tumor without metastasis, rheumatoid arthritis/collagen vascular diseases, coagulopathy, obesity, weight loss, fluid and electrolyte disorders, blood loss anemia, deficiency anemia, alcohol abuse, drug abuse, psychoses, and depression[11].

**Fig 1:**
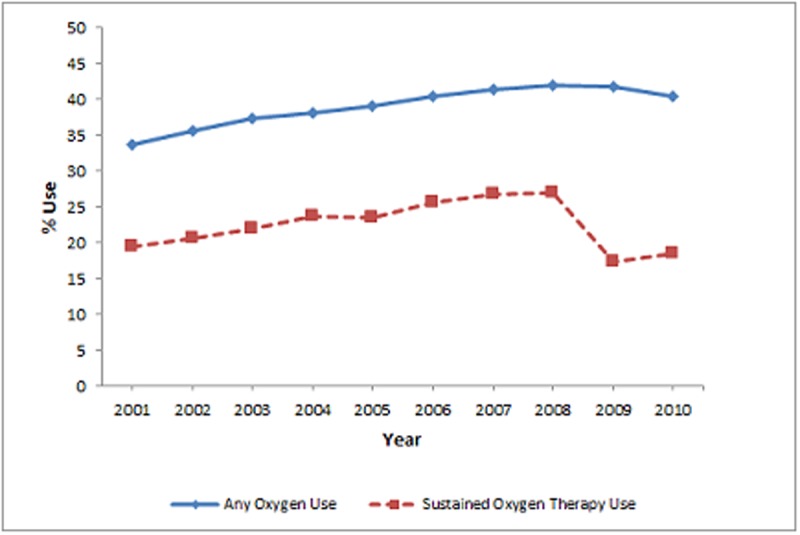
Annual percent of oxygen therapy and Sustained oxygen therapy use from 2001–2010.

**Fig 2:**
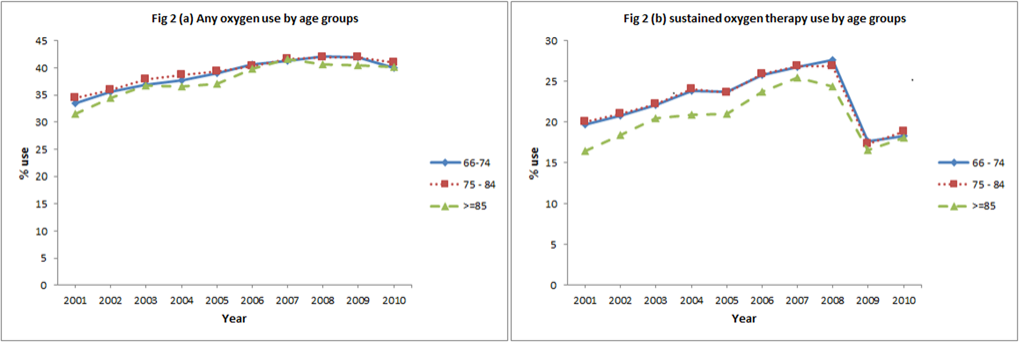
Any oxygen use by age groups, (a), and sustained oxygen therapy use by age group, (b).

**Fig 3:**
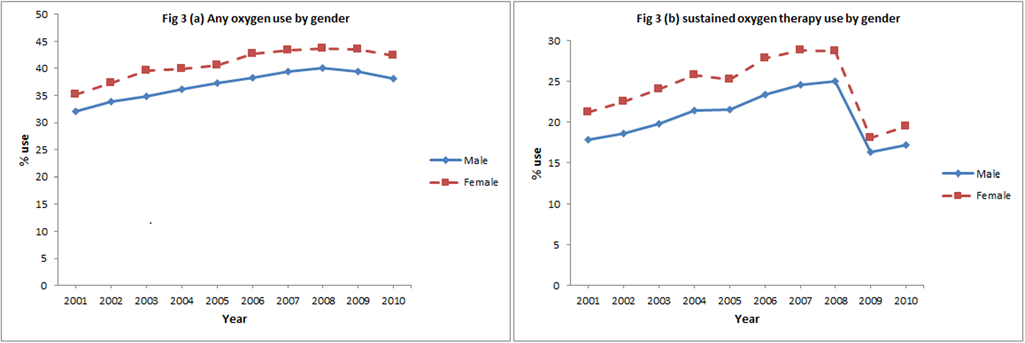
Any oxygen use by gender, (a), and sustained oxygen therapy use by gender, (b).

**Fig 4:**
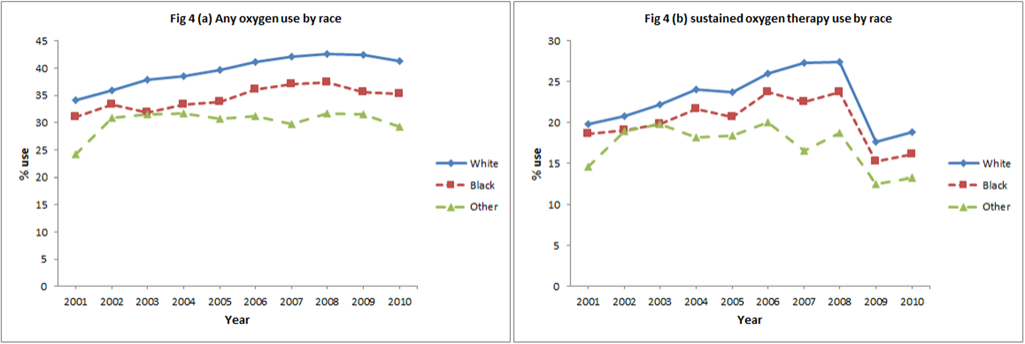
Any oxygen use by race, (a), and sustained oxygen therapy use by race, (b).

**Fig 5:**
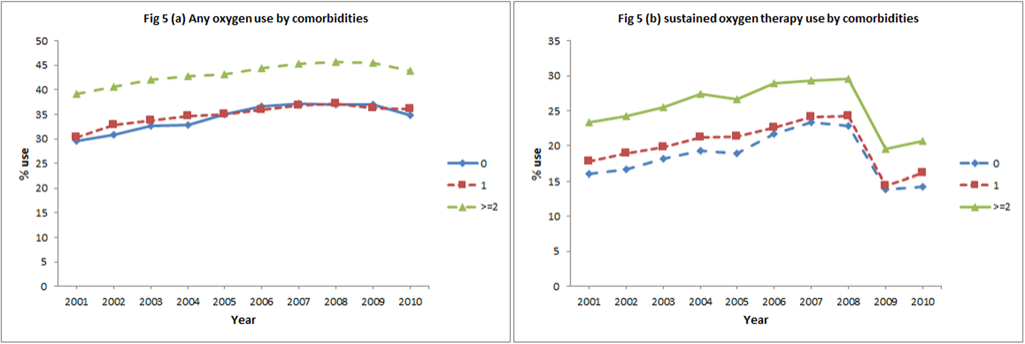
Any oxygen use by comorbidities, (a), and sustained oxygen therapy use by comorbidities, (b).

In the initial eight years, subjects receiving oxygen therapy increased from 33.7% to 41.9% and sustained oxygen therapy increased from 19.5% to 26.9%. After 2008, the percentage of subjects receiving oxygen therapy declined slightly (-1.4%) over the subsequent 2 years while receipt of sustained oxygen therapy declined more steeply (-8.4%). Over 10 year period, a net increase in oxygen therapy (6.8%) and a net decrease in sustained oxygen therapy (-1.0%) was observed (Fig. 1). Figs. 2–5a-b show the annual percent of oxygen therapy and sustained oxygen therapy use over the study period by age, gender, race and number of comorbidities. As shown in the figures, receipt of any oxygen and sustained oxygen therapy was higher in females, non-Hispanic whites and those with ≥2 comorbidities.

Table 2 presents the multivariable analysis of factors associated with oxygen therapy and sustained oxygen therapy receipt in COPD subjects which included 157,150 patients with 329,482 personal years. Factors associated with higher odds of receiving oxygen therapy and sustained oxygen therapy include female gender, non-Hispanic white race, and low socioeconomic status. Compared to 2001, subjects in 2008 had 33% higher odds of receiving oxygen therapy (odds ratio [OR] 1.33, 95% Confidence Interval [CI] 1.29–1.38) and 43% higher odds of receiving sustained oxygen therapy (odds ratio [OR] 1.43, 95% Confidence Interval [CI] 1.37–1.48). After 2008, there was a significant decline in odds of receiving sustained oxygen therapy while the odds of receiving oxygen therapy declined only slightly. A piecewise regression model was performed to examine the annual trend of change in oxygen usage. During 2001–2008, oxygen therapy and sustained oxygen therapy use increased 4.1%/year and 4.4%/year, respectively; during 2008–2010, oxygen therapy and sustained oxygen therapy use decreased 5.8% and 32.1%, respectively.

**Table 2:** Multivariable analysis of odds of Oxygen Therapy and Sustained oxygen therapy use among COPD patients.

	Odds of Any Oxygen Therapy (95% CI)	Odds of Sustained oxygen therapy (95% CI)
**Age**		
66–74	Ref	Ref
75–84	1.03 (1.01–1.06)	1.01 (0.99–1.04)
≥85	0.99 (0.96–1.03)	0.91 (0.87–0.94)
**Gender**		
Female	Ref	Ref
Male	0.87 (0.85–0.89)	0.83 (0.81–0.85)
**Race**		
White	Ref	Ref
Black	0.73 (0.69–0.77)	0.77 (0.73–0.82)
Other	0.58 (0.54–0.62)	0.62 (0.57–0.67)
**Low Socioeconomic Status**		
No	Ref	Ref
Yes	1.25 (1.21–1.29)	1.28 (1.24–1.32)
**Elixhauser Comorbidity**		
0	Ref	Ref
1	1.04 (1.02–1.07)	1.12 (1.09–1.16)
≥2	1.55 (1.52–1.59)	1.59 (1.55–1.64)
**Year**		
2001	Ref	Ref
2002	1.08 (1.05–1.11)	1.05 (1.02–1.09)
2003	1.14 (1.11–1.18)	1.13 (1.09–1.17)
2004	1.17 (1.14–1.21)	1.24 (1.20–1.28)
2005	1.21 (1.18–1.25)	1.21 (1.17–1.26)
2006	1.29 (1.25–1.33)	1.36 (1.31–1.41)
2007	1.32 (1.28–1.37)	1.43 (1.37–1.48)
2008	1.33 (1.29–1.38)	1.43 (1.37–1.48)
2009	1.29 (1.25–1.34)	0.78 (0.75–0.82)
2010	1.22 (1.18–1.26)	0.85 (0.82–0.88)
**Region**		
New England	Ref	Ref
East North Central	1.64 (1.55–1.74)	1.54 (1.44–1.65)
East South Central	1.64 (1.54–1.75)	1.53 (1.42–1.65)
Middle Atlantic	0.98 (0.92–1.05)	1.01 (0.94–1.09)
Mountain	2.86 (2.66–3.07)	2.54 (2.35–2.75)
Pacific	1.39 (1.30–1.48)	1.30 (1.21–1.41)
South Atlantic	1.63 (1.54–1.72)	1.54 (1.45–1.65)
West North Central	1.94 (1.81–2.08)	1.83 (1.69–1.98)
West South Central	1.70 (1.60–1.81)	1.55 (1.44–1.66)

In a sensitivity analysis, we compared ≥6 and ≥11 oxygen claims by calendar year versus a 12 month rolling calendar. Percentage oxygen use with ≥6 oxygen claims in a calendar year and ≥11 oxygen claims in a rolling calendar year are similar (Table 3). Trends in sustained oxygen therapy use are similar regardless of rolling month versus calendar year with the lowest percentage of sustained oxygen therapy noted using ≥11 oxygen claims in a calendar year. Among patients with oxygen claims, 50.3% were evaluated only by PCPs, 33.4% were co-managed by a PCP and a pulmonary specialist and 7.6% were evaluated only by a pulmonary specialist in the one year prior to the first oxygen claim.

**Table 3:** Oxygen use among chronic obstructive pulmonary disease (COPD) patients by calendar year and by rolling 12 months.

		Calendar Year		Rolling 12 Months	
Year	≥1 oxygen claim	≥6 oxygen claims	≥11 oxygen claims	≥6 oxygen claims	≥11 oxygen claims
**2001**	33.7	26.0	19.5	27.6	25.4
**2002**	35.6	27.6	20.6	29.5	27.3
**2003**	37.3	28.8	22.0	30.9	28.6
**2004**	38.1	30.4	23.7	32.1	29.7
**2005**	39.0	31.1	23.4	32.5	30.0
**2006**	40.5	32.5	25.6	34.1	31.8
**2007**	41.4	33.7	26.7	35.3	33.0
**2008**	41.9	34.0	26.9	35.6	33.2
**2009**	41.7	27.7	17.3	31.5	26.3
**2010**	40.5	27.8	18.5	31.4	26.5

Oxygen use determined by number of defined from Healthcare Common Procedure Coding System (HCPCS) code E1390-E1392 from the DME file.

During the study period there were 65,587 (32.5%) unique COPD subjects who received prescription for oxygen. Of beneficiaries with COPD receiving oxygen, 4,070 (6.2%) participated in pulmonary rehabilitation within a year after the first oxygen claim. Survival analysis showed that the median survival of females was 6 months longer than males (51 months versus 45 months) after initiation of sustained oxygen therapy.

## Discussion

Our study describes oxygen therapy and sustained oxygen therapy in the Medicare population over time. From 2001 to 2010, claims of oxygen therapy increased while sustained oxygen therapy decreased.

Prior landmark trials describe long term oxygen therapy (LTOT) loosely. The MRC study characterized LTOT as 15h/d oxygen use over a 3 year follow up period, until death or study end[2]. The NOTT distinguished between nocturnal (12 hours) and continuous oxygen (≥19 hours) use for a one year follow up period, until death or study end [1]. We define and use the term sustained oxygen therapy as ≥11 claims/calendar year due to inherent limitations determining hours of daily oxygen use in administrative data. Additional differences are noted in the present study. Compared to the MRC and NOTT studies, our cohort age is on average 10 years older, has more comorbidity and over half were women.

The reasons for an increase in oxygen therapy and sustained oxygen therapy during the study period are multiple. Although the prevalence of COPD in the United States remained stable between 1998 through 2009, survival of COPD patients has improved. Particularly, females have longer life expectancies, and since 2007, females affected by COPD outnumbered males, which may account for the rise in oxygen use in females [13–15]. Similar trends were demonstrated in a Danish study examining LTOT over the same time period as our study [16]. The shift in the burden of COPD to females may be due to the rise in smoking rates in females or an increased susceptibility to and severity of COPD due to smoking and pollutants [15, 17–22]. In addition, oxygen use may be increasing because of increased physician awareness of its benefits in COPD, decreased patient perception of oxygen therapy as a stigma, and relative ease of obtaining DME for oxygen therapy.

Our finding of decline in oxygen therapy and particularly a sharp decline in sustained oxygen therapy after 2008 may be attributed to policy changes. Prior studies have shown that over half of oxygen therapy prescriptions do not meet Medicare criteria [23]. Up to 91% of patients qualify for oxygen during an acute exacerbation of COPD despite recommendations that oxygen needs should be assessed in clinically stable patients [16]. The Global Strategy for Diagnosis, Management, and Prevention of COPD (GOLD) guidelines recommend reassessment 4–6 weeks after hospital discharge in patients who initially qualified during a period of clinical instability [9]. However, only 33–65% undergoes reassessment, the majority no longer meet Medicare criteria for oxygen therapy and up to a third require a significant change to their oxygen prescription [16, 23–29]. In 2013, the American College of Chest Physicians (ACCP) and American Thoracic Society (ATS) emphasized that reassessment should occur no later than 90 days after discharge as one of five initiatives outlined in the Choosing Wisely campaign to promote more effective use of health care resources [30].

Rising DME spending and inappropriate use of oxygen therapy prompted several healthcare policies addressing oxygen qualification and reimbursement. Under the Medicare Modernization Act of 2003 the DME Prosthetics, Orthotics, and Supplies (POS) suppliers entered into a competitive bidding program for oxygen therapy contracts which began in 2009. In 2006, as part of the Deficit Reduction Act, a capped rental program began limiting reimbursement of oxygen and supplies at 36 months. The year 2009 marks the first year the rental cap was reached since the program began. Additionally, the Medicare Improvements for Patients and Providers Act (MIPPA) of 2008 guarantees maintenance, service and provision of supplies up to 2 years after the 36 month cap without further reimbursement, and instituted an additional 9.5% reimbursement reduction to home oxygen suppliers beginning 2009 [6, 31]. Although competitive bidding is shown to save on average 42% to Medicare and its beneficiaries, changes in healthcare policy have culminated in a decline in oxygen supplier reimbursement and the decreased number of home oxygen suppliers [6, 32]. Any or a combination of these factors may account for decreased sustained oxygen therapy claims.

Pulmonary rehabilitation is an important non-pharmacologic management of patients with COPD. Historically, use of this modality is very low—1–2% [33, 34]. In our study, 6.2% of beneficiaries were enrolled in pulmonary rehabilitation. This high rate reflects the COPD cohort requiring oxygen therapy which is shown as an independent predictor of pulmonary rehabilitation attendance [35]. Providers should consider enrolling patients qualified for oxygen use in a pulmonary rehabilitation program.

The results of this study may have been influenced by several limitations. First, our cohort was limited to beneficiaries aged ≥66 years with Medicare Parts A and B coverage, and our findings may not be relevant to younger patients or individuals enrolled in HMOs. However, over 1 million Medicare beneficiaries receive sustained oxygen therapy, which represents >80% of all oxygen use [36]. Carrier files provide a reliable record of the care *received*, but not necessarily the care *needed*. For instance, a documented COPD diagnosis does not confirm accuracy of diagnosis. However, claims data using ICD-9 codes for COPD have been validated as reliable for the purpose of extracting information [37]. The use of claims data to study home oxygen use only accounts for a device being prescribed and paid. We have no information on the total duration of oxygen used by the patient in a 24 hour period. This information does not account for an estimated 68% of COPD patients with hypoxemia who do not receive home oxygen, subjects who received but did not use oxygen, or those who were prescribed but never received sustained oxygen therapy [38]. Our definition of sustained oxygen therapy included a minimum duration of oxygen claims so as to exclude intermittent oxygen therapy prescribed for acute exacerbations but may still include oxygen use for frequent exacerbations; additionally, these subjects may have met the definition of sustained oxygen therapy use more than once over the study period. Our study may also include use of sustained oxygen therapy for exertional or nocturnal desaturations, which have not definitively been shown to improve survival. A similar study in Sweden showed that 28% of subjects qualify for oxygen therapy for conditions other than COPD [27]. Due to the nature of the study, we may not be able to provide the true estimates of sustained oxygen therapy use; however, the study provides important information on the magnitude and the trend of oxygen and sustained oxygen therapy use over time.

In summary, we report an increase in oxygen therapy use but a decrease in sustained oxygen therapy in fee-for-service Medicare beneficiaries with COPD from 2001 to 2010. The decrease in sustained oxygen therapy claims may be due to changes in healthcare policy affecting oxygen reimbursement. Current oxygen users tend to be older females with multiple comorbidities.
